# Endocrinological outcomes of pure endoscopic transsphenoidal surgery: a Croatian Referral Pituitary Center experience

**DOI:** 10.3325/cmj.2012.53.224

**Published:** 2012-06

**Authors:** Andreja Marić, Ivan Kruljac, Vatroslav Čerina, Hrvoje Ivan Pećina, Petra Šulentić, Milan Vrkljan

**Affiliations:** 1 Referral Center for Clinical Neuroendocrinology and Pituitary Diseases, Sestre Milosrdnice University Hospital, Zagreb, Croatia; 2 University of Zagreb, School of Medicine, Zagreb, Croatia

## Abstract

**Aim:**

To analyze early remission, complications, and pituitary function recovery after pure endoscopic endonasal transsphenoidal surgery (PEETS), a novel method in pituitary adenoma treatment.

**Methods:**

Testing of all basal hormone values and magnetic resonance imaging (MRI) were performed preoperatively and postoperatively (postoperative MRI only in nonfunctioning adenomas) in 117 consecutive patients who underwent PEETS in the period between 2007 and 2010. The series consisted of 21 somatotroph adenomas, 61 prolactinomas, and 4 corticotroph and 31 nonfunctioning adenomas. Sixty-three were macroadenomas and 54 were microadenomas. Remission was defined as hormonal excess normalization on the seventh postoperative day in functioning adenomas and as normal MRI findings approximately four months postoperatively in nonfunctioning adenomas. The presence of hypogonadism, growth hormone deficiency, and hypothyroidism was assessed on the seventh postoperative day. Hypocortisolism was assessed through necessity for replacement therapy within 18 months postoperatively.

**Results:**

Remission was achieved in 84% of patients: in 100% of microadenoma and 70% of macroadenoma patients (*P* < 0.001, odds ratio [OR], 28.16, 95% confidence interval [CI], 1.61-491.36), respectively. Endocrinological complications occurred in 17.1% of patients: in 9% of microadenoma and 24% of macroadenoma patients (*P* = 0.049, OR, 3.06; 95% CI, 1.03-9.08). Duration of empirical hydrocortisone replacement therapy was significantly shorter in microadenoma patients (*P* < 0.001). Thirty-five percent of preoperatively present hormonal deficiencies improved after the surgery. Between tumor types there were no significant differences in remission, complications, and normal pituitary function recovery.

**Conclusion:**

Patients with microadenomas had higher remission and lower complication rates following PEETS, emphasizing the necessity for early detection and treatment of pituitary adenomas. PEETS is a discussion-worthy method for microprolactinoma treatment.

Transsphenoidal pituitary surgery can be performed by two techniques: microsurgical and endoscopical. Pure endoscopic endonasal transsphenoidal surgery (PEETS) is a minimally invasive technique introduced by Jho in 1993 (1). The first pure endoscopic procedure in our Center was performed in 2004. Our Pituitary Center is a single Croatian institution that routinely uses pure endoscopic technique in pituitary surgery. Some of the PEETS advantages are panoramic vision inside the surgical area, a superior close-up of the anatomy, and an improved working angle (2). Although studies that compare these two techniques are scarce, PEETS is proven to be associated with a decrease in the length of hospital stays and operative times, mean blood loss, immediate postoperative diabetes insipidus, some rhinologic complications, and patient’s pain and discomfort (3,4).

Surgery is a well established first-line treatment for somatotropinomas, corticotropinomas, and nonfunctioning adenomas (5,6). On the other hand, medical therapy with dopamine agonists (DA) is considered to be the first line treatment for prolactinomas (7). However, only 21% of patients remain normoprolactinemic after DA withdrawal (8). Consequently, DA treatment is often life-long, requires intensive long-term follow-up and high costs, and impairs the quality of life (9). Recent studies have reported excellent remission and low complication rates in surgical treatment of microprolactinomas using the classical microsurgical technique (10,11). Pituitary surgery has an additional role in restoring normal pituitary function in patients with hypopituitarism caused by all types of pituitary adenomas. It has been shown that approximately half of hormonal deficiencies caused by pituitary adenoma improve after surgery (12).

The main goal in pituitary surgery is defined as maximal tumor removal with maximal preservation of pituitary function (13). To the best of our knowledge, no study has evaluated remission, endocrinological and anatomical complications, and pituitary function improvement in the same series of patients treated with PEETS. Therefore, we analyzed all basal hormone values and magnetic resonance imaging (MRI) before and after PEETS (postoperative MRI only in nonfunctioning adenomas), as well as early remission, complications, and hypopituitarism improvement according to tumor size and type.

## Patients and methods

### Patients

This study included 117 consecutive patients with pituitary adenoma who underwent PEETS at the Referral Center for Clinical Neuroendocrinology and Pituitary Diseases, Sestre Milosrdnice University Hospital between January 2007 and January 2010. All patients with residual or recurrent adenomas and patients with other tumors of the sellar region were excluded. The series consisted of 36 (31%) male and 81 (69%) female patients, with a mean age of 45.0 ± 17.5 years (range 17-84 years). Sixty-three patients were diagnosed with macroadenomas and 54 with microadenomas. The series consisted of 61 (52.1%) patients with prolactinomas (mean age 33.4 ± 14.2 years), 31 (26.5%) with nonfunctioning adenomas (mean age 52.5 ± 15.4 years), 21 (17.9%) with somatotropinomas (mean age 50.6 ± 16.4 years), and 4 (3.5%) with corticotropinomas (mean age 57.3 ± 5.2 years). All patients underwent the same preoperative and postoperative endocrinological and radiological examination.

### Radiological examination

The site and size of the tumor were evaluated by magnetic resonance imaging (MRI) with and without administration of intravenous contrast agent. We used a 1.5 Tesla MRI according to the standard protocol. It included native T1 and T2-weighted imaging and dynamic T1-weighted imaging after gadolinium-base contrast medium. Tumors up to 10 mm in size were classified as microadenomas and tumors larger than 10 mm as macroadenomas.

### Hormonal assays

Prolactin (PRL) was measured using the flow injection analysis method – reference range for men was 2.0-20.0 μg/L and for women 2.0-30.0 μg/L. Electrochemiluminescence immunoassay was used for measurement of serum cortisol, growth hormone (GH), insulin-like growth factor- I (IGF-I), follicle-stimulating hormone (FSH), luteinizing hormone (LH), and estradiol. Reference range for cortisol in 8 am was 138-800 nmol/L and in 5 pm was 80-488 nmol/L. Reference range for GH was 0.0-5.0 ng/mL and for IGF-I 115-420 ng/mL. Reference range for FSH in follicular phase was 3.5-12.5 IJ/L for women in reproductive age, 25.8-134.8 IJ/L for menopausal women, and 1.5-12.4 IJ/L for men. Reference range for LH in follicular phase was 2.4-12.6 IJ/L, 7.7-58.5 IJ/L for menopausal women and 1.7-8.6 IJ/L for men. Reference range for estradiol was 80-790 pmol/L in follicular phase. Radioimmunoassay method was used for determination of urine-free cortisol and testosterone. Reference range for urine-free cortisol was 72.5-372.0 nmol/24 hours and for testosterone in men between 20 and 50 years of age 8.6-29.0 nmol/L and in men older than 50 years 6.7-25.7 nmol/L. Chemiluminescence immunoassay was used for thyroid- stimulating hormone (TSH), T4, and adrenocorticotrophic hormone (ACTH) measurement. Reference ranges were the following: for TSH 0.4-4.0 mIJ/L, for T4 60-165 nmol/L, and for ACTH 2.0-13.3 pmol/L.

### Preoperative endocrinological evaluation

Concentrations of plasma GH, IGF-I, PRL, ACTH, serum cortisol, 24-hour urinary free cortisol, TSH, thyroid hormones, LH and FSH, testosterone in men, and progesterone and estradiol in women were measured in all patients. Additional testing performed in acromegalic patients consisted of somatostatin test (with GH determination) and bromocriptine test (measuring GH and PRL response), which was used to assess the efficacy of medical treatment in case of surgical failure. Positive somatostatine and bromocriptine tests were defined as normalization of GH serum levels. Bromocriptine and sulpiride tests were performed (with determination of PRL levels) in patients with hyperprolactinemia. Bromocriptine test was used for assessment of response to medical therapy and was defined as normalization of PRL levels after peroral administration of 2.5 mg of bromocriptine. Sulpiride test was used as accessory tool in differentiating the cause of hyperprolactinemia. Positive sulpiride test was defined as 3-fold increase of serum PRL after peroral sulpiride administration. In patients with hypercortisolism, we performed overnight 1 mg dexamethasone suppression test and 5-hour dexamethasone infusion test (with ACTH and cortisol determination). Biochemical criteria for diagnosis of acromegaly were increased GH (>5 ng/mL; reference range 0-5 ng/mL) and IGF-I levels (>420 ng/mL; reference range 115-420 ng/mL). After secondary hyperprolactinemia (including polycystic ovary syndrome) had been excluded, prolactinoma was diagnosed based on the correlation of positive magnetic resonance findings with increased PRL level (>30 ng/mL; reference range 2- 30 ng/mL) and negative sulpiride stimulation test. Elevated or normal plasma ACTH level (reference range 2.0 -13.3 pmol/L), elevated serum cortisol (reference range: 8 am 138-800 nmol/L, 5 pm 80-488 nmol/L), altered diurnal cortisol rhythm, increased 24-hour urinary free cortisol (reference range 72.5-372 nmol/24h), and negative dexamethasone suppression test (morning cortisol above 80 mmol/L) along with partial suppression of cortisol levels after 5-hour dexamethasone infusion were biochemical criteria for Cushing’s disease.

Secondary thyroid hormone deficiency was defined as decreased T4 (<6) and decreased or normal TSH serum levels. Secondary hypogonadism in women was defined as decreased or normal FSH and LH and decreased estradiol, and in men as decreased or normal LH and low testosterone level. Growth hormone deficiency was considered as decreased IGF-I level and adrenal insufficiency was defined as decreased morning serum cortisol and urinary-free cortisol level.

### Surgical indications

Surgical indications were assessed after complete endocrinological testing performed during rounds consisting of endocrinologist, neurosurgeon, and neuroradiologist, taking the patient’s preference into consideration whenever possible. Surgical indications were all in accordance with clinical guidelines. All patients diagnosed with acromegaly, Cushing’s disease, macroprolactinoma, and nonfunctioning macroadenoma underwent surgery. Nonfunctioning microadenomas that manifested with headache and/or were adjacent to cavernous sinus were also treated surgically. Patients with microprolactinomas previously treated with dopamine agonists had classical surgical indications: dopamine agonist intolerance, inability of normalization of PRL hypersecretion, and inability of reducing tumor size. The majority of patients with microprolactinomas were newly diagnosed. All patients with cystic microprolactinomas were treated with PEETS. In others, indication for surgery was patient’s preference.

### Pure endoscopic endonasal transsphenoidal technique

All patients were operated on by the same experienced neurosurgeon (V.C.), who exclusively performs PEETS in our institution. Patient's head was fixated without Mayfield's fixator and the exact position was determined using the x-ray generator. The final decision on which nostril to use was made after the inspection of both nostrils. A 0°, 30°, or 45° rigid endoscope (180-300/4 mm) was used. The endoscope was navigated into the nasal cavity. It passed between the inferomedial part of the middle turbinate and the nasal septum and reached the sphenoethmoidal recess and sphenoidal sinus aperture. We did not resect the middle turbinate, it was gently lateralized but preserved, so normal post-operative middle meatus physiology could be maintained. Posterior part of the vomer (between the sphenoidal rostrum and palatinal crest) was removed in order to approach the sphenoid sinus. The anterior wall of the sphenoidal sinus was opened. Inside the sphenoidal sinus, the anterior sellar wall was localized. Endoscope was then fixated to the holder. The anterior sellar wall and the dura mater were opened with a highspeed drill, Kerrison or Stammberger rongeur. Visualization of the sellar region was initially performed with a 0° endoscope. Following tumor resection, both 0° and angled (30° and 45°) endoscopes were placed into the surgical cavity to explore for any residual tumor. The sellar region was reconstructed with adipose tissue. Fibrin glue was used in cases of intraoperative liquorrhea. The aim of the treatment was total tumor removal without peri- or postoperative complications, including hypopituitarism.

### Pathohistological evaluation

Due to relatively high costs of immunohistochemistry, the final diagnosis was confirmed by immunostaining in all prolactinoma patients with serum PRL level lower than 150 µg/L and in acromegaly patients with GH levels lower than 5 ng/mL and IGF-I levels lower than 600 ng/mL Immunohistochemistry was performed in all patients with Cushing’s disease.

### Postoperative endocrinological evaluation and criteria

Hydrocortisone replacement therapy in daily dose of 30 mg was empirically started on first postoperative day and continued for the following 6 weeks. Blood samples for endocrinological retesting were taken on the seventh postoperative day. Endocrinological evaluation included measurement of the hormone that was preoperatively in excess and measurement of serum cortisol and urinary free cortisol, T4, TSH, IGF-I, estradiol in women, and testosterone in men. Results were evaluated 6 weeks postoperatively. Replacement therapy for other hormone deficiencies was initiated based on the results of endocrinological testing. Hydrocortisone replacement therapy was adjusted or discontinued six weeks, three, six or twelve months after surgery based on the results of endocrinological testing and preoperative tumor size. Insulin tolerance test was performed in the majority of patients with macroadenomas prior to replacement discontinuation, and peak cortisol level of 550 nmol/L was considered to be a sufficient response. If urinary free cortisol level with 10 mg of hydrocortisone was above 200 nmol/24 hours or morning serum cortisol level without hydrocortisone replacement was above 300 nmol/L, replacement therapy was discontinued without insulin tolerance test. Replacement therapies continuing for more than 18 months were considered to be permanent.

Postoperative remission was defined as normalization of hormonal excess in secreting adenomas on the seventh postoperative day. Biochemical remission of acromegaly was defined as a decrease of IGF-1 < 420 ng/mL and GH<5.0 ng/mL. Since oral glucose tolerance test administration requires time and is costly, it was not included in our protocol. Remission for prolactinomas was defined as PRL<30 ng/mL for female and PRL<20 for male patients. Remission of Cushing's disease was defined as a normal postoperative 24-hour urinary free cortisol, normal ACTH and cortisol level, restorement of cortisol diurnal rhythm or continuous glucocorticoid hormone replacement therapy. Postoperative remission in NF adenomas was defined as an absence of residual tumor tissue on MR imaging 3 to 6 months after surgery.

Complications were divided into two groups: anatomical (nasal bleeding, cranial nerve palsy, cerebrospinal fluid leak) and endocrinological (anterior and posterior pituitary dysfunctions). Newly developed hypogonadism, growth hormone deficiency, and hypothyroidism were assessed on the seventh postoperative day, and newly developed adrenal insufficiency was defined as necessity for permanent hydrocortisone therapy.

Improvement of hypogonadism, GH deficiency, and hypothyroidism was assessed on the seventh postoperative day. Postoperative improvement of hypogonadism was defined as normalization of serum testosterone levels in men and serum estradiol levels in women. Improvement of growth hormone deficiency and hypothyroidism was defined as normalization of IGF-I and T4 serum levels. Since hydrocortisone replacement had been administered empirically to all patients, improvement of adrenal insufficiency was defined as discontinuation of hydrocortisone replacement therapy within 18 months after surgery.

### Statistical analysis

Continuous variables are shown as mean ± standard deviation and range. Variables with categorical values are presented as percentages. Quantitative variables (patients’ characteristics) were compared using independent samples *t* test since the variables showed normal distribution. Fisher exact test was used to analyze qualitative variables (influence of tumor size and tumor type on remission and complication rates). Odds ratio (OR) was used to evaluate the association strength between tumor size, remission, and complications. For statistical analysis, we used SAS for Windows software, version 9.1.3. (SAS Inc., Cary, NC, USA) licensed to Zagreb University School of Medicine. *P* value <0.05 was considered significant.

## Results

Patients with microadenomas were significantly younger than patients with macroadenomas (*P* < 0.001) (Table 1). No other significant differences were found in baseline patients’ characteristics.

**Table 1:** Characteristics of 117 patients who underwent pure endoscopic endonasal surgery

	No. (%) of patients with				
Characteristic	prolactinoma (n = 61)	omatotropinoma (n = 21)	corticotropinoma (n = 4)	nonfunctioning adenoma (n = 31)	total (n = 117)
Sex:					
male	13 (21)	9 (43)	2 (50)	12 (39)	36 (31)
female	48 (79)	12 (57)	2 (50)	19 (61)	81 (69)
Tumor size:					
microadenoma (<1 cm)	39 (64)	9 (43)	3 (75)	3 (10)	54 (46)
macroadenoma (>1 cm)	22 (36)	12 (57)	1 (25)	28 (90)	63 (54)
Age in years (mean ± standard deviation):					
male	41.6 ± 17.5	47.8 ± 20.3	58.0 ± 4.2	50.3 ± 16.8	47.9 ± 17.1
female	32.7 ± 14.6	52.3 ± 12.9	56.0 ± 7.1	53.0 ± 16.1	42.0 ± 17.7
P*^†^	0.130	0.648	0.764	0.620	0.104
Microadenoma	28.6 ± 8.7	47.6 ± 13.1	55.7 ± 5.0	43.6 ± 20.2	35.8 ± 13.6
Macroadenoma	41.0 ± 18.5	50.6 ± 16.4	61	53.3 ± 15.5	49.5 ± 17.3
P*^‡^	0.009	0.674	-	0.324	<0.001

*Unpaired *t* test.

†Difference between men and women.

‡Difference between patients with microadenoma and macroadenoma.

Overall postoperative remission in our series was achieved in 83.8% (98/117) of patients: 69.8% (44/63) with macroadenomas and 100% with microadenomas (54/54). Remission was achieved in 80.6% of patients with nonfunctioning adenomas and in 84.9% of those with functioning adenomas. Tumor size significantly influenced surgical outcome (Table 2). Patients diagnosed with microadenoma had greater chance for remission after surgery (OR, 28.168; 95% CI, 1.615-491.364). Tumor type did not significantly influence surgical outcome (*P* = 0.073). Remission was achieved in 90.2% (55/61) of patients with prolactinomas. All patients with microadenomas met the criteria for early remission (39/39), unlike 72.7% (16/22) of those with macroadenomas. Remission was achieved in 15/21 patients with acromegaly (71.4%); 6/12 patients with macroadenomas (50%) and 9/9 patients with microadenomas (100%). Remission was achieved in all three patients with Cushing’s disease caused by microadenoma. In one patient with surgical failure, bilateral adrenalectomy led to sufficient disease control. Neuroradiological criteria for remission were obtained in 80.6% (25/31) of patients with nonfunctioning adenomas, in all three microadenoma patients, and in 78.6% (22/28) of macroadenoma patients. Patients with microprolactinomas and microsomatotropinomas had significantly higher remission rates.

**Table 2:** Comparison of remission and endocrinological complications after pure endoscopic endonasal transsphenoidal surgery in patients with microadenomas and in those with macroadenomas

	No (%) of patients with											
	prolactinoma			somatotropinoma			nonfunctioning adenoma			total		
Variable	microadenoma (N = 39)	macroadenoma (N = 22)	P*	microadenoma (N = 9)	macroadenoma (N = 12)	P*	microadenoma (N = 3)	macroadenoma (N = 28)	P*	microadenoma (N = 54)	macroadenoma (N = 63)	P*
Remission	39 (100)	16 (73)	0.001	9 (100)	6 (50)	0.019	3 (100)	22 (79)	1.000	54 (100)	44 (70)	0.001
Endocrinological complications	1 (3)	2 (9)	-	2 (22)	4 (33)	-	0 (0)	9 (32)	0.537	5 (9)	15 (24)	0.049

*Fisher exact test.

We observed 20.5% (24/117) of complications. Endocrinological complications were recorded in 17.1% of patients (20/117). Newly developed permanent adrenal insufficiency was recorded in 9.4% (11/117) of patients, hypogonadism in 1.7% of patients (2/117), and growth hormone deficiency in only one patient. Transient diabetes insipidus occurred in 3.4% (4/117) of patients and permanent diabetes insipidus in 1.7% (2/117) of patients. Tumor size significantly influenced the occurrence of endocrinological complications (Table 2). Patients with microadenomas had significantly lower complication rates (OR, 3.063; 95% CI, 1.032-9.087). Recovery of adrenal function after surgery was also significantly faster in patients with microadenomas (Table 3). There were no significant differences between complication rates and tumor type. Anatomical complications were less common (3.4%, 4/117): 1.7% (2/117) of patients had nasal bleeding, 0.8% (1/117) had sixth cranial nerve palsy, and only 0.8% (1/117) had cerebrospinal fluid leak. All complications occurred in patients with macroadenomas except for one patient with nasal bleeding. There were no major complications such as meningitis, carotid artery lesions, or death.

**Table 3:** Dynamics of adrenal function recovery after pure endoscopic endonasal transsphenoidal surgery in patients with microadenomas and macroadenomas

	No (%) of patients with											
	prolactinoma			somatotropinoma			nonfunctioning adenoma			total		
Adrenal function recovery after (months):	microadenoma (N = 39)	macroadenoma (N = 22)	P*	microadenoma (N = 9)	macroadenoma (N = 12)	P*	microadenoma (N = 3)	macroadenoma (N = 28)	P*	microadenoma (N = 54)	macroadenoma (N = 63)	P*
six	29 (74)	9 (41)	0.014	5 (56)	2 (17)	0.159	3	4 (14)	0.008	38 (70)	13 (21)	<0.001
eighteen	10 (26)	10 (45)	0.157	3 (33)	6 (50)	0.660	-	11 (39)	-	13 (24)	31 (49)	0.007
Permanent insufficiency	0 (0)	3 (14)	0.043	1 (11)	4 (33)	0.620	-	13 (47)	-	2 (6)	20 (32)	<0.001

*Fisher exact test.

Endocrinological complications were recorded in three prolactinoma patients (4.9%). One case of permanent adrenal insufficiency was recorded in a patient with macroadenoma. Two patients (one microadenoma and one macroadenoma patient) experienced transient diabetes insipidus. Significantly higher proportion of patients with microadenomas recovered their adrenal function within six months after surgery (Table 3). Six endocrinological complications occurred in patients with somatotropinoma. Hypogonadism occurred in one microadenoma and one macroadenoma patient. Hypocortisolism occurred in one microadenoma patient and three macroadenoma patients. Nasal bleeding occurred in one macroadenoma patient and transient sixth cranial nerve palsy occurred in one invasive macroadenoma patient. Transient diabetes insipidus and hypocortisolism occurred in one patient with Cushing’s disease due to macroadenoma. Nine endocrinological (29.0%) and three anatomical complications (9.7%) were recorded in patients with nonfunctioning adenomas. There were no complications in microadenoma group, while in macroadenoma group, growth hormone deficiency occurred in one patient (3.6%), hypocortisolism in five (17.9%), transient diabetes insipidus in one (3.6%), and permanent diabetes insipidus in two (7.1%) patients. There was one case of cerebrospinal fluid leak resolved after lumbar drainage on the third postoperative day and two cases of nasal bleeding (delayed bleeding from small branch of sphenopalatine artery treated with posterior nasal tamponade in one patient and minor nasal bleeding on the second postoperative day treated with unilateral packing in the second patient).

**Figure 1:**
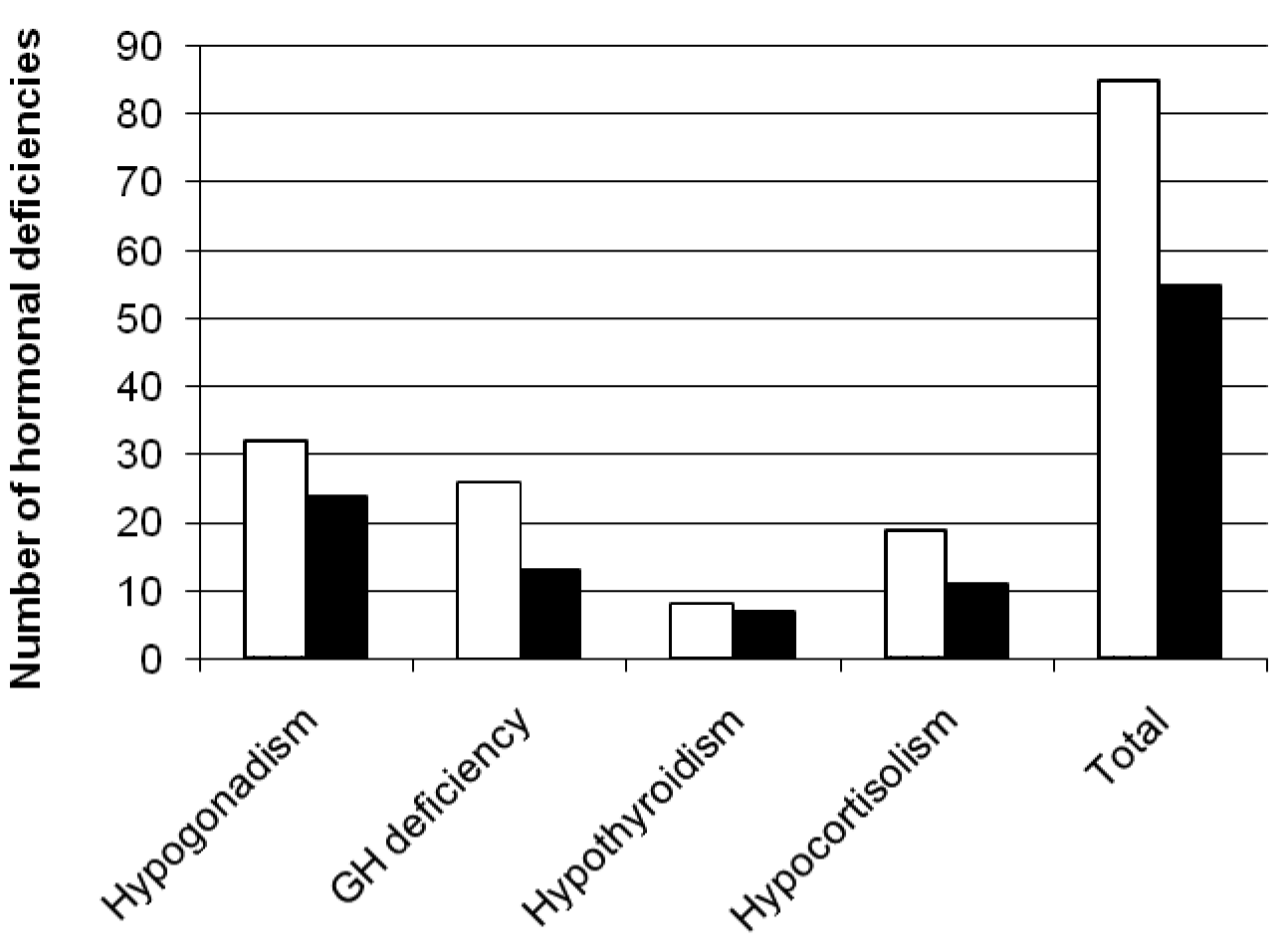
Hormonal deficiencies before (open bars) and after (closed bars) pure endoscopic endonasal transsphenoidal surgery in patients with macroadenomas.GH – growth hormone.

Since there were no hormonal deficiencies in patients with microadenomas, hypopituitarism improvement was analyzed in macroadenoma patients. There were 85 hormonal deficiencies recorded in 63 patients. Improvement was recorded in 35.3% (30/85) of hormonal deficiencies. Preoperatively, hypogonadism was recorded in 50.8% (32/63), growth hormone deficiency in 41.3% (26/63), adrenal insufficiency in 30.2% (19/63), and hypothyroidism in 12.7% (8/63) of patients. Hormonal deficiency improved in 26.7% (8/30) of patients with hypogonadism, 50.0% (13/26) with growth hormone deficiency, 42.1% (8/19) with adrenal insufficiency, and 12.5% with hypothyroidism (1/8) (Figure 1). Hypogonadism was recorded in 10/22 prolactinoma patients and improved in two patients. GH deficiency was recorded in 5/22 and improved in three patients, while hypocortisolism was recorded in five and improved in three patients. Hypogonadism was present in three macrosomatotropinoma patients and improved in one patient. Hypocortisolism was recorded in two and improved in one patient. A total of 60 hormonal deficiencies were recorded in 28 patients with nonfunctioning macroadenomas preoperatively. There were 19 cases of hypogonadism, which improved in 5 (26.3%) patients. Growth hormone deficiency improved in 47.6% (10/21), adrenal insufficiency in 33.3% (4/12), and hypothyroidism in 12.5% (1/8) of patients.

## Discussion

Our study presents one of the largest series of patients treated with PEETS by single neurosurgeon in such a short time period and is the first to analyze remission, endocrinological and anatomical complications, and hypopituitarism improvement in the same series of patients treated with PEETS. In all other studies on PEETS, the authors focused only on remission (the extent of tumor removal), anatomical complications and diabetes insipidus, constantly ignoring anterior pituitary function. In addition, this is the largest series of patients with prolactinomas initially treated with PEETS.

Overall postoperative remission in our series was achieved in 83.8% (98/117) of patients; 69.8% with macroadenomas (44/63) and 100% with microadenomas (54/54). Remission was achieved in 80.6% of patients with nonfunctioning adenomas and in 84.9% with functioning adenomas. It is extremely difficult to compare these results with other studies due to a wide variety of remission criteria. However, a metaanalysis on remission and anatomical complications consisting of eight studies reported an overall remission rate of 78% (14). Similar results were reported in a more recent study, which obtained an overall remission rate of 79.3% (83% for nonfunctioning and 76.3% for functioning adenomas) after a median follow-up of 61.5 months (15). Studies that evaluated initial remission in functioning adenomas reported the rates of 63%-75.6% (16,17). Our results are slightly better than those reported in all previous studies, possibly due to a larger proportion of patients with microprolactinomas in our series, which might have a more favorable outcome.

A total of 20.5% (24/117) of complications was recorded, endocrinological in 17.1% (20/117) and anatomical in 3.4% of cases. Complication rates (especially anatomical) were slightly lower when compared with Gondim’s series, in which 26.9% (81/301) of complications was observed, 17.9% endocrinological and 8.97% anatomical (18). Anatomical complication rates in our series are similar to those reported by Zhang et al (3%, 21/678) (19). We observed low rates of postoperative cerebrospinal fluid leaks (0.8% in our series compared to 2.6% reported by Gondim). In the metaanalysis conducted by Tabee, postoperative cerebrospinal fluid leak occurred in 2% and permanent diabetes insipidus in 1% of patients (14). Lower rates of cerebrospinal fluid leak in our series are not in accordance with previous studies reporting on higher rates of cerebrospinal fluid leak after PEETS (16). A total of 17.1% of endocrinological complications in our series is in accordance with Gondim’s study (18), which is the single reported study on endocrinological complications after PEETS. Endocrinological complications differ tremendously between hormonal deficiencies in our series. We recorded far more adrenal insufficiencies than other hormonal deficiencies. This is possibly due to different methodology for the analysis of adrenal insufficiency, which was analyzed indirectly through the necessity for hydrocortisone treatment over the first 18 months after surgery, unlike other hormonal deficiencies that were analyzed on the seventh postoperative day. This different approach toward the assessment of adrenal axis is due to empirical hydrocortisone therapy, which needed to be administered routinely to all patients prior and three months after the surgery, since there are no reliable predictors of sufficient adrenal function (20). Additional explanation of this discrepancy might be in the fact that routine hydrocortisone therapy by itself might potentially influence adrenal axis recovery via inhibition of corticotropin secretion. Our results confirm that microadenoma patients recover their pituitary function faster than macroadenoma patients. The majority of microadenoma patients require hydrocortisone replacement only within the first six months after surgery.

It is considered that pituitary deficiencies in patients with pituitary adenomas are caused by compression and destruction of the normal pituitary gland by the expanding mass and possibly due to focal necrosis after compression of the portal circulation (12,21). In Webb's series, in 48% of patients with hypopituitarism at least one hormone deficiency was improved (12). In another series, growth hormone deficiency recovered in 15%-47%, hypogonadism improved in 29%-32%, adrenal insufficiency in 38-5, and hypothyroidism in 13%-57% of the cases (8,12,21,22). In our series, 35.3% of hormonal deficiencies improved after surgery: in 26.7% of patients with hypogonadism, 50.0% with growth hormone deficiency, 42.1% with adrenal insufficiency, and 12.5% with hypothyroidism. However, our results are hardly comparable with those in previous studies, due to different periods after surgery in which hormonal status was assessed. Hypopituitarism progressively improves after surgery (23). Arafah and Webb evaluated recovery one to six months postoperatively, unlike in our study where hypogonadism, GH deficiency, and hypothyroidism improvement were assessed on the seventh postoperative day (12,21). But nevertheless, improvement rates in our series were only slightly inferior to theirs. These data emphasize the role of increased intrasellar pressure in the pathophisiology of hypopituitarism caused by pituitary adenoma. Recovery of adrenal insufficiency in our series was assessed within 18 months after surgery, similar to Berg’s study (23), in which hypocortisolism was evaluated by insulin-tolerance test 12 months after surgery. Recovery rate of 42.1% in our series is comparable to that reported by Berg (recovery in 55% of patients 12 months postoperatively), despite the difference in criteria in these two studies (23).

Since we were the first to analyze the outcomes of PEETS in large series of patients with prolactinomas, we wish to discuss the role of PEETS in prolactinoma treatment. DA are first-line treatment for prolactinomas (7). However, up to one third of patients treated with bromocriptine experienced systemic side-effects, 12% did not tolerate the drug in therapeutic doses, and 5%-10% showed minimal or no response to treatment (24,25). Cabergoline has been shown to be more effective in normalizing PRL levels, with significant reduction of adverse effects (26), but its use is limited in developing countries due to relatively high costs. Transsphenoidal surgery improved substantially over the last 20 years. In Turner’s study on microsurgical treatment of 32 microprolactinomas published twelve years ago, remission rates were 78% with 13 (40%) hormonal deficiencies developed after the surgery (27). In the most recent study, Babey reported remission rates of 94% in 34 small prolactinomas with one case of newly developed hypogonadism (complete hormonal status was not determined) (11). This is similar to Kreutzer’s study with remission rates of 91.3% in 56 microprolactinoma patients and overall endocrinological complication rates of 8.8% in 171 prolactinoma patients (micro-, macro-, giant adenomas) (10). We achieved remission in 100% (39/39) of patients with microprolactinomas, along with only one case of transient diabetes insipidus, without permanent hormonal deficiencies. Endocrinological complications were recorded in 3 (4.9%) macroprolactinoma patients, with one case of permanent hypocortisolism (1.6%). Our results are slightly superior to all previously published data, suggesting that PEETS might be a valuable alternative to DA therapy. There is no doubt that medical treatment of prolactinomas is the first-line treatment. However, since the majority of prolactinomas are detected in the stage of microadenoma (7,28), treatment with PEETS in experienced specialized centers might be a reasonable alternative to DA treatment.

Our study has some limitations: the data are largely descriptive and it is a non-randomized, single institutional study. Also, due to a small number of patients, statistical analysis could not be performed between certain groups. In conclusion, patients with microadenomas have higher remission and lower complication rates following PEETS, emphasizing the necessity for early detection and treatment of pituitary adenomas. PEETS is a discussion-worthy method for microprolactinoma treatment.

## References

[1] Jho HD, Carrau RL (1997). Endoscopic endonasal transsphenoidal surgery: experience with 50 patients.. J Neurosurg.

[2] Dehdashti AR, Ganna A, Karabatsou K, Gentili F (2008). Pure endoscopic endonasal approach for pituitary adenomas: early surgical results in 200 patients and comparison with previous microsurgical series.. Neurosurgery.

[3] Strychowsky J, Nayan S, Reddy K, Farrokhyar F, Sommer D (2011). Purely endoscopic transsphenoidal surgery versus traditional microsurgery for resection of pituitary adenomas: systematic review.. J Otolaryngol Head Neck Surg..

[4] Rotenberg B, Tam S, Ryu WH, Duggal N (2010). Microscopic versus endoscopic pituitary surgery: a systematic review.. Laryngoscope.

[5] Arnaldi G, Angeli A, Atkinson AB, Bertagna X, Cavagnini F, Chrousos GP (2003). Diagnosis and complications of Cushing’s syndrome: a consensus statement.. J Clin Endocrinol Metab.

[6] Melmed S, Colao A, Barkan A, Molitch M, Grossman AB, Kleinberg D (2009). Guidelines for acromegaly management: an update.. J Clin Endocrinol Metab.

[7] Casanueva FF, Molitch ME, Schlechte JA, Abs R, Bonert V, Bronstein MD (2006). Guidelines of the Pituitary Society for the diagnosis and management of prolactinomas.. Clin Endocrinol (Oxf).

[8] Dekkers OM, Lagro J, Burman P, Jřrgensen JO, Romijn JA, Pereira AM (2010). Recurrence of hyperprolactinemia after withdrawal of dopamine agonists: systematic review and meta-analysis.. J Clin Endocrinol Metab.

[9] Kars M, van der Klaauw AA, Onstein CS, Pereira AM, Romijn JA (2007). Quality of life is decreased in female patients treated for microprolactinoma.. Eur J Endocrinol.

[10] Kreutzer J, Buslei R, Wallaschofski H, Hofmann B, Nimsky C, Fahlbusch R (2008). Operative treatment of prolactinomas: indications and results in a current consecutive series of 212 patients.. Eur J Endocrinol.

[11] Babey M, Sahli R, Vajtai I, Andres RH, Seiler RW (2011). Pituitary surgery for small prolactinomas as an alternative to treatment with dopamine agonists.. Pituitary.

[12] Webb SM, Rigla M, Wagner A, Oliver B, Bartumeus F (1999). Recovery of hypopituitarism after neurosurgical treatment of pituitary adenomas.. J Clin Endocrinol Metab.

[13] Chandler WF, Barkan AL. Pituitary surgery: techniques. In: Swearingen B, Biller BMK, editor. Diagnosis and management of pituitary adenomas. Totowa: HumanaPress; 2008. p. 289-302.

[14] Tabaee A, Anand VK, Barron Y, Hiltzik DH, Brown SM, Kacker A (2009). Endoscopic pituitary surgery: a systematic review and meta-analysis.. J Neurosurg.

[15] Gondim JA, Schops M, de Almeida JP, de Albuquerque LA, Gomes E, Ferraz T (2010). Endoscopic endonasal transsphenoidal surgery: surgical results of 228 pituitary adenomas treated in a pituitary center.. Pituitary.

[16] D'Haens J, Van Rompaey K, Stadnik T, Haentjens P, Poppe K, Velkeniers B (2009). Fully endoscopic transsphenoidal surgery for functioning pituitary adenomas: a retrospective comparison with traditional transsphenoidal microsurgery in the same institution.. Surg Neurol.

[17] Hofstetter CP, Shin BJ, Mubita L, Huang C, Anand VK, Boockvar JA (2011). Endoscopic endonasal transsphenoidal surgery for functional pituitary adenomas.. Neurosurg Focus.

[18] Gondim JA, Almeida JP, Albuquerque LA, Schops M, Gomes E, Ferraz T (2011). Endoscopic endonasal approach for pituitary adenoma: surgical complications in 301 patients.. Pituitary.

[19] Zhang Y, Wang Z, Liu Y, Zong X, Song M, Pei A (2008). Endoscopic transsphenoidal treatment of pituitary adenomas.. Neurol Res.

[20] Karaca Z, Tanriverdi F, Atmaca H, Gokce C, Elbuken G, Selcuklu A (2010). Can basal cortisol measurement be an alternative to the insulin tolerance test in the assessment of the hypothalamic-pituitary-adrenal axis before and after pituitary surgery?. Eur J Endocrinol.

[21] Arafah BM (1986). Reversible hypopituitarism in patients with large nonfunctioning pituitary adenomas.. J Clin Endocrinol Metab.

[22] Marazuela M, Astigarraga B, Vicente A, Estrada J, Cuerda C, Garcıa-Urıa J (1994). Recovery of visual and endocrine function following transsphenoidal surgery of large non-functioning pituitary adenomas.. J Endocrinol Invest.

[23] Berg C, Meinel T, Lahner H, Mann K, Petersenn S (2010). Recovery of pituitary function in the late-postoperative phase after pituitary surgery: results of dynamic testing in patients withpituitary disease by insulin tolerance test 3 and 12 months after surgery.. Eur J Endocrinol.

[24] Boyd A (1995). Bromocriptine and psychosis: a literature review.. Psychiatr Q..

[25] Webster J (1996). A comparative review of the tolerability profiles of dopamine agonists in the treatment of hyperprolactinaemia and inhibition of lactation.. Drug Saf.

[26] Webster J, Piscitelli G, Polli A, Ferrari Cl, Ismail I, Scanlon MF (1994). A comparison of cabergoline and bromocriptine in the treatment of hyperprolactinemic amenorrhea. Cabergoline Comparative Study Group.. N Engl J Med.

[27] Turner HE, Adams CB, Wass JA (1999). Trans-sphenoidal surgery for microprolactinoma: an acceptable alternative to dopamine agonists?. Eur J Endocrinol.

[28] Vrkljan M, Matovinovic M, Maric A, Bekc M, Zah T, Resetic J (2006). Incidence of pituitary tumors in the human population of Croatia.. Coll Antropol.

